# An Interaction Effect Analysis of Thermodilution-Guided Hemodynamic Optimization, Patient Condition, and Mortality after Successful Cardiopulmonary Resuscitation

**DOI:** 10.3390/ijerph18105223

**Published:** 2021-05-14

**Authors:** Enikő Kovács, Valéria Anna Gyarmathy, Dávid Pilecky, Alexandra Fekete-Győr, Zsófia Szakál-Tóth, László Gellér, Balázs Hauser, János Gál, Béla Merkely, Endre Zima

**Affiliations:** 1 Department of Anaesthesiology and Intensive Therapy, Semmelweis University, H-1428 Budapest, Hungary; hauser.balazs@med.semmelweis-univ.hu (B.H.); gal.janos@med.semmelweis-univ.hu (J.G.); 2 Medical Department, EpiConsult Biomedical Consulting and Medical Communication Agency, Dover, DE 19901, USA; agyarma1@jhu.edu; 3 Department of Epidemiology, Johns Hopkins University, Baltimore, MD 21218, USA; 4 Department of Internal Medicine III, Klinikum Passau, 94032 Passau, Germany; pileckyd@gmail.com; 5 Anaesthetics Department, Hillingdon Hospital, London UB 8 3NN, UK; alexandra.feketegyor@nhs.net; 6 Heart and Vascular Center, Semmelweis University, H-1428 Budapest, Hungary; zsofia.szakaltoth@gmail.com (Z.S.-T.); geller.laszlo@med.semmelweis-univ.hu (L.G.); merkely.bela@med.semmelweis-univ.hu (B.M.); zima.endre@gmail.com (E.Z.)

**Keywords:** cardiac arrest, resuscitation, hypothermia, hemodynamic monitoring, thermodilution, mortality

## Abstract

Proper hemodynamic management is necessary among post-cardiac arrest patients to improve survival. We aimed to investigate the effects of PiCCO™-guided (pulse index contour cardiac output) hemodynamic management on mortality in post-resuscitation therapy. In this longitudinal analysis of 63 comatose patients after successful cardiopulmonary resuscitation cooled to 32–34 °C, 33 patients received PiCCO™, and 30 were not monitored with PiCCO™. Primary and secondary outcomes were 30 day and 1 year mortality. Kaplan–Meier curves and log-rank tests were used to assess differences in mortality among the groups. Interaction effects to disentangle the relationship between patient’s condition, PiCCO™ application, and mortality were assessed by means of Chi-square tests and logistic regression models. A 30 day mortality was significantly higher among PiCCO™ patients, while 1 year mortality was marginally higher. More severe patient condition per se was not the cause of higher mortality rate in the PiCCO™ group. Patients in better health conditions (without ST-elevation myocardial infarction, without cardiogenic shock, without intra-aortic balloon pump device, or without stroke in prior history) had worse outcomes with PiCCO™-guided therapy. Catecholamine administration worsened both 30 day and 1 year mortality among all patients. Our analysis showed that there was a complex interaction relationship between PiCCO™-guided therapy, patients’ condition, and 30 day mortality for most conditions.

## 1. Introduction

Sudden cardiac arrest is one of the leading causes of deaths in Europe [1,2]. The so-called chain of survival describes the main steps, which play a crucial role in improving the survival of cardiac arrest: early recognition of cardiac arrest and call for help; early cardiopulmonary resuscitation (CPR); early defibrillation; and proper post-resuscitation therapy [3]. It needs to be emphasized that the chain is as strong as its weakest link. If all links were properly strengthened, the survival of this population would increase. All patients requiring hospitalization after the return of spontaneous circulation (ROSC) would benefit from an appropriate post-cardiac arrest therapy and hemodynamic management.

Physicians face several challenges during post-resuscitation therapy at the intensive care unit (ICU). Post-cardiac arrest syndrome occurring after ROSC contains reperfusion-ischemic injury, post-cardiac arrest brain injury, post-cardiac arrest myocardial dysfunction and the precipitating pathology itself that caused cardiac arrest [4]. The prevention and treatment of secondary brain damage and hemodynamic management are co-dependent, and both elements play an important role in post-cardiac arrest therapy [5]. On the one hand, target temperature management (TTM) with target temperature of 32–36 °C is applied as a neuroprotective tool in comatose patients surviving cardiac arrest [6,7,8]. On the other hand, TTM and particularly mild hypothermia may influence the circulatory system in a negative manner leading to bradycardia, arrhythmias, increased systemic vascular resistance, polyuria, and corresponding hypovolemia [9,10]. Post-cardiac arrest syndrome has also a plenty of factors affecting the hemodynamics harmfully [4]. Moreover, a proper hemodynamic management is crucial to keep a satisfactory cerebral perfusion to prevent further cerebral deterioration [11]. Therefore, advanced hemodynamic monitoring may be useful in post-resuscitation therapy. However, there is no evidence about which hemodynamic parameters should be monitored and which monitoring method should be used. Usually, electrocardiogram (ECG), oxygen saturation, invasive arterial blood pressure, respiratory rate, urine output, and blood-gas analysis are monitored routinely in the ICU.

The PiCCO™ (pulse index contour cardiac output) monitoring system (Pulsion Medical Systems, Munich, Germany)-guided therapy uses transpulmonary thermodilution and pulse contour analysis to determine hemodynamic parameters [12]. A cold liquid bolus is applied via a central venous catheter passing through various thoracic compartments, and a peripheral arterial thermodilution catheter (mostly inserted into the femoral superficial artery) detects the temperature curve, calculating cardiac output with the modified Stewart–Hamilton equation [13]. In addition, a continuous cardiac output monitoring by recording the pulse pressure wave can also be used in PiCCO™. Calculated hemodynamic parameters give further information about the circulatory system: the global end diastolic index (GEDI) reflects preload, and the systemic vascular resistance index (SVRI) represents afterload, while the extravascular lung water index (ELWI) gives information about pulmonary edema, and the global ejection fraction (GEF) reflects cardiac systolic function.

PiCCO™ was shown to provide better monitoring capacities in critically ill patients with necrotizing pancreatitis [14], sepsis [15], and in cardiac surgery [16]. It was also assessed during post-cardiac arrest therapy and therapeutic hypothermia (32–34 °C), showing that thermodilution measurements are precise in patients following ROSC, even if a lower temperature is used [17]. In addition, a set of triplicate and higher volume (e.g., at least 10 mL) injections was recommended to be applied in order to achieve the best precision [17]. As such, the European Society of Intensive Care Medicine recommends the use of cardiac output monitoring (and transpulmonary thermodilution) in patients with severe shock not responding to initial treatment [18]. However, to our knowledge there is no assessment regarding whether PiCCO™-monitoring-guided therapy actually improves mortality outcome during post-resuscitation therapy.

Therefore, the aim of our study was to investigate the effects of PiCCO™-guided hemodynamic management on 30 day and 1 year mortality in comatose patients treated with TTM after ROSC. More specifically, we focused on disentangling the relationship between PiCCO™-application, the patients’ condition, and mortality by assessing it as interaction effects.

## 2. Materials and Methods

### 2.1. Study Design

This was a quasi-feasibility retrospective longitudinal chart review analysis of PiCCO™ use among a small real-world patient population. Study participants were all patients (N = 254) who successfully underwent CPR after either OHCA (out-of-hospital cardiac arrest) or IHCA (in-hospital cardiac arrest) between January 2008 and January 2015 at Semmelweis University Heart and Vascular Center, which is the largest and highest-volume cardiovascular center in the country.

Patient assignment to PiCCO™-monitoring followed a real-world systematic consecutive treatment allocation protocol as follows: the Center has only one PiCCO™ system for 10 beds altogether, and if it was available, then it was assigned to the first admitted patient. Any consecutive patient received treatment without PiCCO™, until PiCCO™ was available again. At that point, the next consecutive patient received PiCCO™ monitoring and guidance to treatment.

Institutional medical records and charts were reviewed to estimate the in-hospital management in the first 72 h after admission. Information related to pre-hospital emergency care by OHCA patients was obtained from the records of emergency medical service and Utstein reports.

The Semmelweis University Regional and Institutional Committee of Science and Research Ethics approved our study (approval number: 19/2019). The written informed consent requirement was waived due to the retrospective nature of the study.

### 2.2. Patients

**Figure 1:**
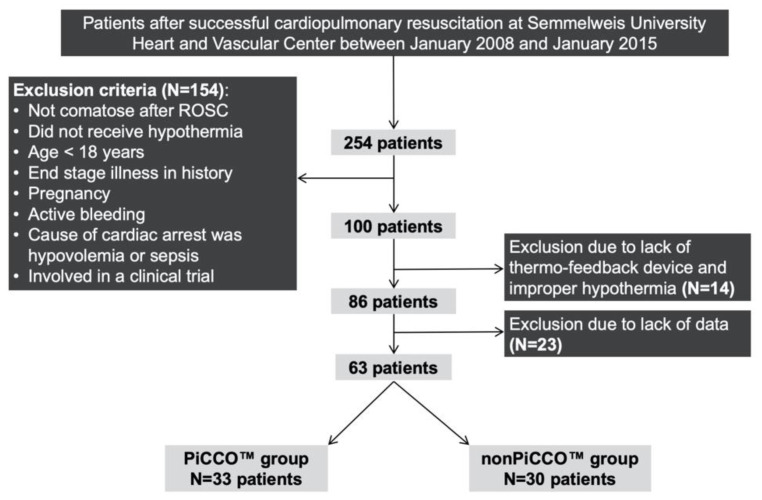
The selection of study population and eligibility of patients. A total of 254 patients after successful cardiopulmonary resuscitation were screened, and 63 were included into the study based on the inclusion and exclusion criteria. *N: number of patients; PiCCO™: pulse index contour cardiac output.*

We included in our analysis those comatose patients who were cooled to 32–34 °C for 24 h after ROSC on the basis of the actual ERC (European Resuscitation Council) guidelines [19], were older than 18 years, had no end-stage illness in history, were not pregnant, had no active bleeding, whose cause of cardiac arrest had a probable cardiac origin, and were not involved in a clinical trial. In addition, only patients cooled with the Blanketrol III™ (Cincinatti SubZero Products, Cincinatti, USA) thermo-feedback device were enrolled into the study—those patients whose temperature management was applied with ice packs and/or physical cooling were excluded because target temperatures could not be reached in most of these cases. Twenty-three patients were excluded from the study due to lack of data. After the sampling process depicted in Figure 1, the final study sample included 63 patients (33 who received and 30 who did not receive PiCCO™ monitoring).

### 2.3. Initial Therapy

Post-resuscitation therapy and TTM were initiated as soon as possible after the admission of OHCA-patients and after ROSC of IHCA-patients. All patients received standardized critical care according to our institutional protocol. An acute coronarography was performed in each case, which was followed by percutaneous coronary intervention (PCI) and/or intra-aortic balloon pump (IABP) insertion, if indicated. All patients were treated in the ICU at the acute phase of the assessment. We upheld mechanical ventilation until the patients fulfilled extubation criteria.

Oxygen saturation, electrocardiogram, invasive arterial blood pressure, central venous pressure, diuresis, blood-gas parameters, and serum lactate level were monitored for all patients. An echocardiography was performed after the admission to the ICU to assess the heart function. If it was available, the basic hemodynamic monitoring was augmented with PiCCO™ monitor (Pulsion Medical System, Munich, Germany); thermodilution measurements were applied at least every 12 h during the first 48 h after the initiation of cooling. We measured the following variables: cardiac index (CI: L/min/m^2^), systemic vascular resistance index (SVRI: dyn.sec.cm^−5^), global end-diastolic volume index (GEDI: mL/m^2^), extravascular lung water index (ELWI: mL/kg/m^2^), and global ejection fraction (GEF: %). If patients had IABP, their device was paused for the time of thermodilution measurement.

**Figure 2:**
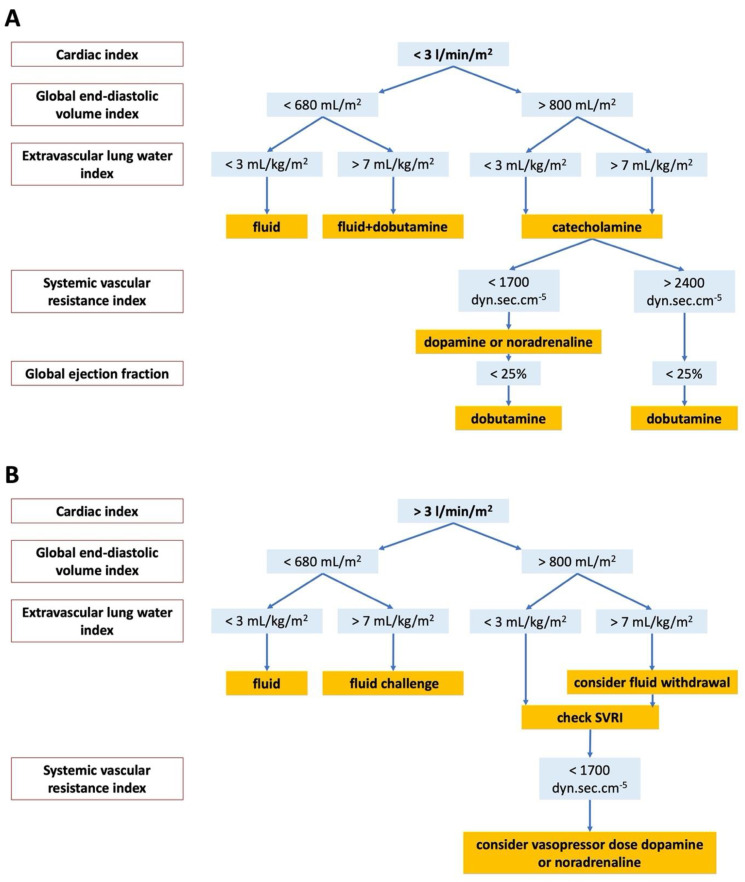
(**A**). Hemodynamic management of patients in PiCCO™ group in the case of low (<3 L/min/m^2^) cardiac index. (**B**). Hemodynamic management of patients in PiCCO™ group in the case of normal or high (>3 L/min/m^2^) cardiac index. SVRI: systemic vascular resistance index.

Fluid, vasopressor, and inotrope therapy were accomplished by monitoring heart rate (HR), mean arterial pressure (MAP), central venous pressure, diuresis, and lactate levels for patients without PiCCO™. We used the goal parameters given by the actual ERC guidelines [20]. Dopamine at a vasopressor dose or noradrenaline were administered as vasopressors after fluid challenge if MAP was still lower than 65 mmHg or diuresis fell below 1 mL/kg/hour. Fluid challenge was administered even in the case of elevated lactate level referring to low perfusion of the peripheral tissues. Dobutamine was used if positive inotropic agent was required based on the echocardiography at admission. The hemodynamic management was guided by PiCCO™ parameters and the principles of therapy decision tree of Pulsion Medical System [21] were applied for PiCCO™ patients. Figure 2A,B show the detailed hemodynamic management of PiCCO™ monitored patients.

A combination of catecholamines was applied in the case of therapy-resistant hemodynamic instability in both groups. As an alternative inotrope levosimendan was administered beside vasopressor treatment based on clinical judgement of the treating physician if long-standing critical low cardiac performance was present.

TTM was divided into three phases: induction of cooling, maintenance phase, and rewarming. During the induction of TTM 30 mL/kg crystalloids were given to the patients, and the previously mentioned thermo-feedback device was used. The temperature in maintenance phase was upheld with the same device. After 24 h of hypothermia, rewarming was achieved by passively trying to keep a 0.25 °C/h rewarming speed until normothermia was reached. Prevention and control of fever still played an important role in the first 72 h of the assessment. Patients received a combination of intravenous benzodiazepine and opioid as sedation during hypothermia. Their core temperature was measured with an esophageal thermometer.

Additionally, patient data extracted included the patients’ age, gender, previous health conditions affecting cardiovascular system (diabetes, hypertension, stroke, hyperlipidemia, and acute myocardial infarction), circumstances of CPR (time between collapse and ROSC, if the patient was on monitor at the time of collapse, if basic life support was performed, and initial rhythm) and important steps of therapy after ROSC (presence of cardiogenic shock (CS), ST segment elevation and non-ST segment elevation myocardial infarction (STEMI and NSTEMI), frequency of acute PCI, frequency of IABP insertion, ejection fraction after ROSC, and necessity of levosimendan administration after ROSC).

To express the adequacy of PiCCO™-guided therapy, we present patients’ changes in temperature during TTM (Figure S1), hemodynamic parameters (Figures S2–S4), changes in serum lactate levels during TTM (Figure S5), and catecholamine dosing (Figures S6–S8) in Supplement.

### 2.4. Patient Outcomes

Primary patient outcome was defined as mortality after 30 days. Secondary patient outcome was defined as mortality after 1 year. All patient follow-up and mortality data were obtained at least up till 1 year after admission based on the health insurance records of the National Health Insurance Fund of Hungary, which contains accurate and valid information on the vital records of the entire population of Hungary. Patients’ death as a hard endpoint was defined as passivation of the healthcare ID in the national records. Patient outcomes were obtained by means of the patients’ unique healthcare ID. Once patient data were matched with the vital records, patient IDs were anonymized for further data management and analysis.

### 2.5. Statistical Analysis

First, three sets of bivariate statistical tests were performed: we assessed differences between (1) the PiCCO and non-PiCCO groups (yes vs. no), (2) mortality after 30 days (yes vs. no) and (3) mortality after 1 year (yes vs. no). Mann–Whitney U tests were used for continuous variables, and Chi-square test or (in case of small sample sizes) Fisher’s exact test was applied for categorical variables. Additionally, Kaplan–Meier curves and log-rank tests for significance were used as longitudinal data to assess differences in mortality between PiCCO and non-PiCCO groups and for those variables where mortality was at least marginally significant using the categorical Chi-square analysis. Given the significant or marginally significant differences between the study groups including the patients’ severity and other characteristics, it is difficult to assess how the hemodynamic management guided by PiCCO™ could affect outcome of the cardiac arrest patients. Therefore, interaction effects were explored (candidate variable vs. PiCCO™ use vs. mortality) using the same statistical methods as in the bivariate analysis for those variables that had at least marginal associations with both PiCCO™ use and mortality. Additionally, logistic regression analysis was performed using the interaction terms as dummy variables. If there were zero cell sizes, then dummy categories were combined with non-zero cells. In a first set of logistic regression models, all interaction dummy variables were included, and in a second set of models, only statistically significant dummies stayed.

Continuous variables are described with median values and their corresponding interquartile range, and categorical data are described as percentages. Not more than 10% of the data were missing; we performed multiple imputation using the k-nearest neighbor algorithm to replace variables with missing values.

We identified *p* < 0.05 for statistical significance and *p* < 0.2 and *p* ≥ 0.05 for marginal significance. Data management and statistical analysis were performed using TIBCO STATISTICA v13.4 (Tibco Software Inc., Palo Alto, CA, USA), and figures were created using GraphPad Prism version 5.0 (GraphPad Software, La Jolla, CA, USA).

## 3. Results

### 3.1. Characteristics of Patients, PiCCO™ Use, 30 Day and 1 Year Mortality

Patient characteristics are shown in Table 1. Altogether, 52% of the patients received PiCCO™, 38% died after 30 days, and 57% died after 1 year.

**Figure 3:**
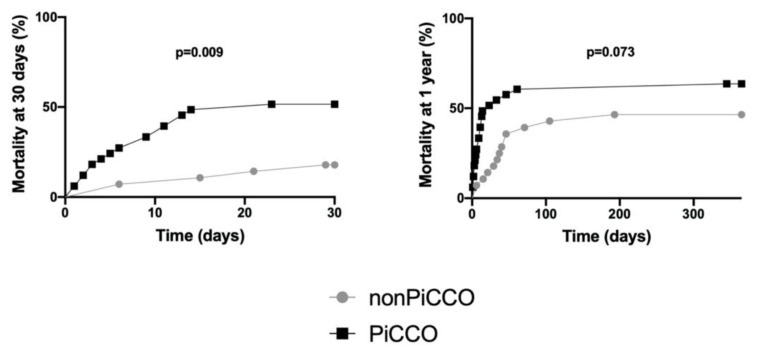
Cumulative incidence of 30 day and 1 year mortality by PiCCO status. Kaplan–Meier curves and log-rank tests were performed. *p*: level of significance; and PiCCO: pulse index contour cardiac output.

Patients with PiCCO™ application were significantly more likely to die after 30 days and marginally more likely to die after 1 year than non-PiCCO™ patients (Figure 3).

**Figure 4:**
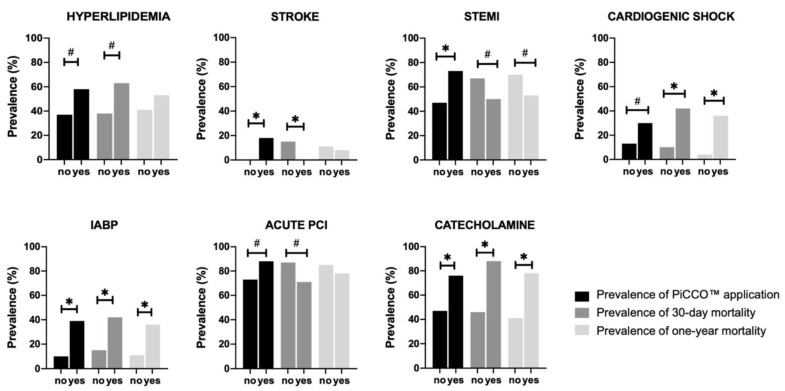
Comparison of PiCCO use, 30 day mortality, and 1 year mortality by patient condition characteristics. Chi-square test was performed. ∗: *p* < 0.05; #: *p* < 0.2; PiCCO: pulse index contour cardiac output; IABP: intra-aortic balloon pump; PCI: percutaneous coronary intervention; and STEMI: ST-elevation myocardial infarction.

As Figure 4 and Supplement Table S1 show, at least a marginal difference for both PiCCO™ application and either 30 day or 1 year mortality was found among patients with hyperlipidemia; prior history of stroke; STEMI as a cause of cardiac arrest; and PCI, CS, IABP, and catecholamine treatment after ROSC. Additionally, males, patients with prior history of hypertension, and those with IHCA were significantly more likely to receive PiCCO™, and patients with non-shockable initial rhythm were significantly more likely to die both after 30 days and 1 year.

### 3.2. Interaction Effects between 30 Day Mortality, PiCCO™ Use and Patient Characteristics

**Figure 5:**
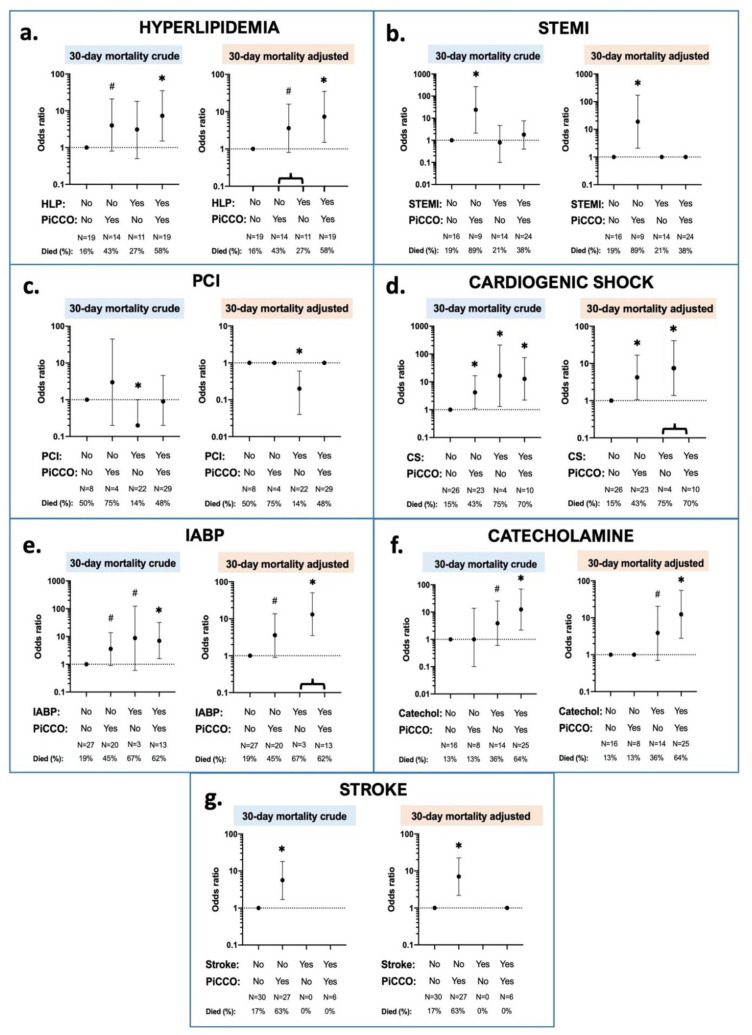
Interaction effects between PiCCO™-application, 30 day mortality, and patient condition characteristics. Patient conditions are depicted in the different subfigures; (**a**) hyperlipidemia; (**b**) STEMI at admission; (**c**): PCI treatment; (**d**): cardiogenic shock at admission; (**e**): IABP insertion; (**f**): catecholamine administration; (**g**): stroke in the prior history. Crude and adjusted logistic regressions were performed. In the crude models, all the interaction term dummy variables were included as separate variables. In the adjusted models, non-significant dummies were combined. ∗: *p* < 0.05; #: *p* < 0.2; catechol: catecholamine; CS: cardiogenic shock; HLP: hyperlipidemia; IABP: intra-aortic balloon pump; N: number of patients; PCI: percutaneous coronary intervention; PiCCO: pulse index contour cardiac output; and STEMI: ST-elevation myocardial infarction.

Figure 5 visualizes the multivariate interaction effects between 30 day mortality, PiCCO™ use, and patient characteristics, statistically controlled for the effects of the subgroups. Accordingly, patients with either PiCCO™ or hyperlipidemia were marginally more likely to die at day 30 than patients with neither PiCCO™ nor hyperlipidemia, while patients with both PiCCO™ and hyperlipidemia were significantly more likely. Moreover, patients with PiCCO™ but no STEMI were significantly more likely to die than patients with no PiCCO™ or patients with both PiCCO™ and STEMI. Additionally, patients with cardiogenic shock regardless of PiCCO™ were significantly much more likely to die than patients with neither cardiogenic shock nor PiCCO™, and patients with PiCCO™ but not cardiogenic shock were significantly more likely. Furthermore, patients with PiCCO™ but no IABP were marginally more likely to die than patients with neither IABP nor PiCCO™, and patients with IABP regardless of PiCCO™ were significantly more likely. In addition, patients receiving catecholamine treatment after ROSC but no PiCCO™ were marginally more likely to die than patients with no catecholamine, and patients with both catecholamine and PiCCO™ were significantly more likely. Moreover, patients with no stroke but with PiCCO™ application were significantly more likely to die than patients with neither stroke nor PiCCO™ or patients with both stroke and PiCCO™. Furthermore, higher mortality was seen in patients with PCI but without PiCCO™, compared to patients without PCI regardless of PiCCO™ or patients with both PCI and PiCCO™.

### 3.3. Interaction Effects between 1 Year Mortality, PiCCO™ Use and Patient Characteristics

**Figure 6:**
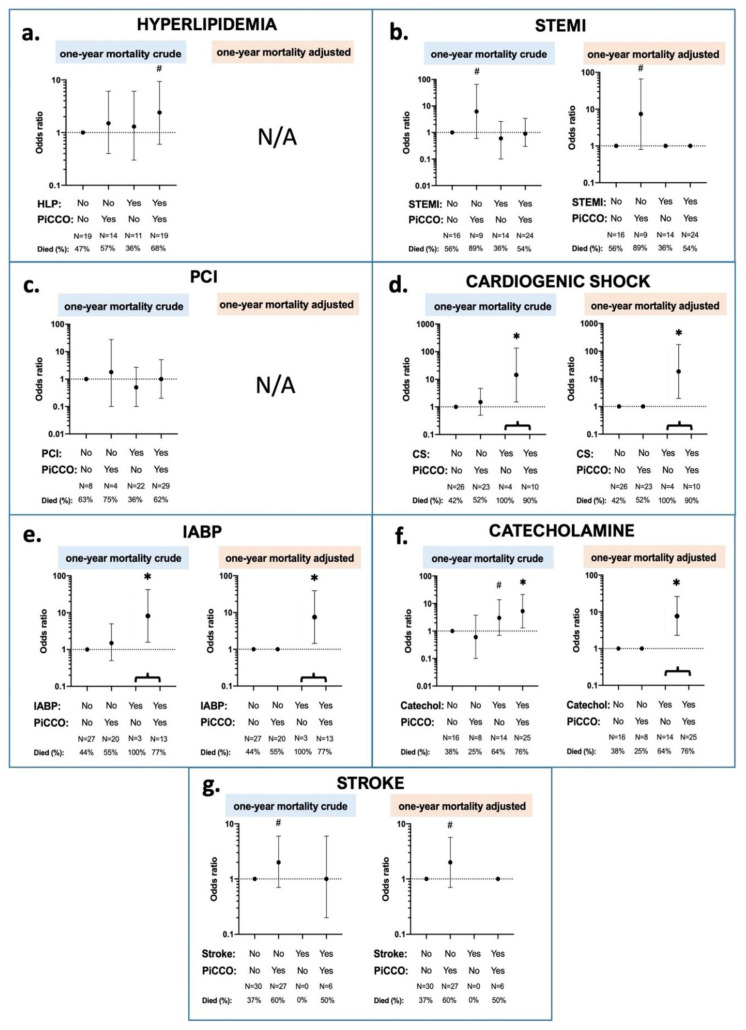
Interaction effects between PiCCO™-application, 1 year mortality, and patient condition characteristics. Patient conditions are depicted in the different subfigures; (**a**) hyperlipidemia; (**b**) STEMI at admission; (**c**): PCI treatment; (**d**): cardiogenic shock at admission; (**e**): IABP insertion; (**f**): catecholamine administration; (**g**): stroke in the prior history. Crude and adjusted logistic regressions were performed. In the crude models, all the interaction term dummy variables were included as separate variables. In the adjusted models, non-significant dummies were combined. ∗: *p* < 0.05; #: *p* < 0.2; catechol: catecholamine; CS: cardiogenic shock; HLP: hyperlipidemia; IABP: intra-aortic balloon pump; N: number of patients; PCI: percutaneous coronary intervention; PiCCO: pulse index contour cardiac output; and STEMI: ST-elevation myocardial infarction.

Multivariate interaction effects between 1 year mortality, PiCCO™ use, and patient characteristics, statistically controlled for the effects of the subgroups, are shown in Figure 6. Patients with PiCCO™ but no STEMI were marginally more likely to die than patients without PiCCO™ regardless of STEMI or patients with both PiCCO™ and STEMI. Additionally, patients receiving catecholamines after ROSC regardless of PiCCO™ were significantly more likely to die than patients who did not receive catecholamine treatment. Moreover, patients with PiCCO™ application but without stroke in past history were marginally more likely to die than patients with both stroke and PiCCO™ or patients with neither stroke nor PiCCO™. Additionally, although no interaction was found for CS, PiCCO™ and 1 year mortality, neither for IABP, PiCCO™, and 1 year mortality, CS and IABP were independent predictors of mortality. Finally, once controlled for subgroups, the interactions between hyperlipidemia, PiCCO™, and 1 year mortality, as well as between PCI, PiCCO™, and 1 year mortality were no longer statistically significant.

## 4. Discussion

To our knowledge, there are no published studies regarding the association between PiCCO™-guided therapy and survival in post-cardiac arrest treatment. We found five interaction patterns between patients’ condition and being monitored with PiCCO™ with regard to mortality after 30 days, which distilled down to three interaction patterns by the end of the first year. As such, our analysis presents a valuable insight into the nuances of advanced hemodynamic monitoring and hemodynamic management during post-cardiac arrest therapy.

The uniqueness of our study is that we disentangled the interaction effects between PiCCO™ application, mortality, and patients’ condition in order to elucidate the potential cause of decayed survival rate in PiCCO™ monitored patients. We found that there was a complex interaction between the use of PiCCO™ and both 30 day and 1 year mortality, depending on the medical condition of the patient.

We identified five groups regarding 30 day mortality. In the first group, having a condition and receiving PiCCO™ meant increased risk compared to either not having the condition or not receiving PiCCO™, as in the case of hyperlipidemia. Specifically, 30 day mortality in patients who both had hyperlipidemia and also received PiCCO™ was higher than among those who either had no hyperlipidemia or received no PiCCO™.

In the second group, not having the condition but receiving PiCCO™ meant increased risk compared to either not having the condition or having the condition regardless of PiCCO™, as in the case of STEMI and stroke. Specifically, 30 day mortality in patients without STEMI (without stroke, respectively) who also received PiCCO™ was higher than among those who either had no STEMI (no stroke, respectively) and received no PiCCO™, or had STEMI (stroke, respectively) regardless of PiCCO™.

In the third group, having the condition or receiving PiCCO™ meant increased risk compared to not having the condition and not receiving PiCCO™, as in the case of CS and IABP. Specifically, 30 day mortality in patients who had either CS (IABP, respectively) or received PiCCO™ was higher than among those who neither had CS (IABP, respectively) nor received PiCCO™.

In the fourth group, not having the condition or receiving PiCCO™ meant increased risk compared to having the condition and not receiving PiCCO™, as in the case of PCI. Specifically, 30 day mortality in patients who had either no PCI or received PiCCO™ was higher than among those who had PCI and did not receive PiCCO™.

In the fifth group, having the condition regardless of PiCCO™ meant increased risk compared to not having the condition, as in the case of receiving catecholamine. Specifically, 30 day mortality in patients who received catecholamine was higher than among those who did not receive catecholamine, regardless of PiCCO™.

Furthermore, we identified three groups regarding 1 year mortality. In the *first group*, there was no difference in 1 year mortality either regarding PiCCO™ application or regarding the presence of the condition, as in the case of hyperlipidemia in prior history or treatment with PCI after ROSC.

In the second group, having the condition regardless of PiCCO™ meant increased risk compared to not having the condition, as in the case of IABP, CS, and catecholamine administration. Specifically, the 1 year mortality was higher in patients treated with IABP (having CS, receiving catecholamine, respectively) than among patients without these conditions regardless of PiCCO™.

In the third group, not having the condition but receiving PiCCO™ meant increased risk compared to either not having the condition or having the condition regardless of PiCCO™, as in the case of STEMI and stroke. Specifically, 1 year mortality in patients without STEMI (without stroke, respectively) who also received PiCCO™ was higher than among those who either had no STEMI (no stroke, respectively) and received no PiCCO™, or had STEMI (stroke, respectively) regardless of PiCCO™.

Adequate hemodynamic management is one of the key elements in post-cardiac arrest therapy. Patients after successful CPR may experience a global ischemic-reperfusion injury and myocardial depression, as parts of post-cardiac arrest syndrome, leading to hemodynamic instability after ROSC [4,22]. Furthermore, the precipitating cause of cardiac arrest itself may result in a deterioration of hemodynamic parameters. It is also well known that TTM has an influence on hemodynamics by several pathways. A decrease in heart rate and cardiac output, an increase in systemic vascular resistance, and hypovolemia caused by raised diuresis may be present as consequences of lower body temperature [23,24,25,26]. As the path leading to hemodynamic instability in post-cardiac arrest syndrome is multifactorial, there is a requirement of proper monitoring tools and proper hemodynamic goal parameters to guide the therapy of these patients.

Despite the complexity of the circulatory effects in post-cardiac arrest period, there is no clear evidence and therefore guidelines about exactly which parameters should be monitored, which goal parameters should be kept during patients’ management, and which monitoring tools should be used to guide the treatment [26,27,28,29,30]. The current ERC guidelines recommend targeting a mean arterial pressure to achieve a satisfactory urine output (1 mL/kg/h) and a decreasing or normal serum lactate level, considering the patient’s habitual blood pressure, the cause of cardiac arrest, and the severity of probable cardiac dysfunction [5]. Moreover, the guidelines suggest that additional cardiac output monitoring may help to guide therapy in hemodynamically unstable patients [5]. However, there is no evidence that cardiac output measuring affects outcome in this patient group.

Transpulmonary thermodilution and pulse contour analysis are applied during PiCCO™ monitoring, which allow intermittent and continuous measurements of cardiac output. Furthermore, several additional measured and calculated parameters can be estimated beside cardiac output reflecting preload, cardiac function, and the vascular tone [12,31]. However, pulmonary artery catheter is the gold standard of thermodilution-based cardiac output measurement [32]. PiCCO™ is less invasive, and it is less influenced by respiratory fluctuations and has a longer dwell time [33,34]. The European Society of Intensive Care Medicine suggests the application of artery pulmonary catheter only in refractory shock with concomitant right-ventricular failure [18]. PiCCO™ was found to be effective in the evaluation of hemodynamic situations in critically ill patients, leading to a faster decision making [35,36]. The PiCCO™-guided hemodynamic management shortened the duration of vasoactive therapy, mechanical ventilation, and ICU stay among elderly patients with cardiogenic shock after acute myocardial infarction [37]. However, there is a lack of evidence regarding the effect of PiCCO™ monitoring system and PiCCO™-guided therapy on mortality in post-cardiac arrest treatment—and this is why our study findings are unique and much needed.

The results of the interaction effect analysis between PiCCO™ application, mortality, and patients’ characteristics show that more severe patient condition per se was not the cause of higher mortality rate in the PiCCO™ group. Moreover, patients in better health conditions (without STEMI, without cardiogenic shock, without the need of IABP support or without stroke in prior history) had worse outcomes against PiCCO™-guided therapy. Our finding supports the literature, as the European Society of Intensive Care Medicine recommends the use of cardiac output monitoring and the application of transpulmonary thermodilution in patients with severe shock not responding to initial therapy [18].

In addition, significantly more catecholamines were administered overall in the PiCCO™ group tailored by the results of the cardiac performance and resistance measurements. Furthermore, a tendency of higher doses of dopamine, dobutamine, and noradrenaline could be observed during TTM in PiCCO™ monitored patients with a significant difference in noradrenaline dosing after rewarming. It is well known that long-term catecholamine administration itself may worsen the outcome [38,39]. The casual relationship raises further questions: Does the advanced hemodynamic monitoring provide benefits to patients who do not have severe shock or are they overtreated based on the PiCCO™ measurements? It was shown that noradrenaline improves tissue perfusion and cardiac output in severely hypotensive patients; however, in the lack of hypotension it may impair microvascular perfusion [40,41].

Furthermore, we need to point out that the injured brain after successful CPR frequently has an impaired autoregulation resulting in the MAP dependence of cerebral blood flow [11,29]. This fact raises the question if the monitoring of cardiac output and advanced hemodynamic parameters is the proper way to guide post-cardiac arrest therapy or the much simpler standard MAP measurement and the clinical judgement of tissue perfusion give enough information.

We suggest applying PiCCO™ in post-cardiac arrest therapy in selected cases based on our results. Moreover, further prospective studies are needed to clarify which patient groups benefit from cardiac output monitoring and thermodilution methods after successful CPR.

Another important finding of our study was that catecholamine administration worsened both 30 day and 1 year mortality in this patient group. It is important to identify the causality of worse survival regarding catecholamine dosage: is the catecholamine itself worsening the outcome, or are the people receiving catecholamine sicker? On the one hand, the side effects of catecholamine usage (arrhythmias, increased cardiac oxygen consumption, splanchnic hyper-fusion, etc.) may worsen outcome, and only the least necessary amount of catecholamine, if any, should be administered [38]. On the other hand, a delay in catecholamine therapy or an improper hemodynamic stabilization may lead to tissue hypoperfusion and deterioration in patients’ condition [42]. Further investigation is required to explain the effects and characteristics of catecholamine treatment in post-cardiac arrest therapy.

Our study has a number of limitations, most of which are related to its retrospective nature and small sample size. Patients were enrolled into the study non-randomly, and the analysis was based on retrospectively collected data. The grouping of patients was assigned based on the availability of a PiCCO™ monitor. Although the baseline characteristics of the groups have been proven to be well-balanced, statistically only one interaction effect analysis was available between the three variable groups. The lack of possibility to adjust for other factors may have left some confounding elements that should be addressed in a bigger study population.

The improper evaluation of PiCCO™ measurements should represent an additional limitation of our study. However, the trends of catecholamine and vasoactive dosage followed the changes of specific hemodynamic parameters, showing the adequacy of therapy. In addition, serum lactate levels decreased in parallel during and after TTM in both the PiCCO™ and non-PiCCO™ groups. Although a local protocol was available regarding hemodynamic management at our ICU, we cannot rule out some therapy-related and treating physician-preference based differences, since treatment in the ICU cannot always be standardized.

Another limitation may be that many factors including cause of cardiac arrest, arrest time, and duration of cardiopulmonary resuscitation may be different between IHCA and OHCA, and therefore, patients and their outcomes may be different. We performed a statistical analysis comparing IHCA and OHCA patients and found no significant differences, which may be due to the very small number of patients in the IHCA group. Therefore, we consider that there was no statistical necessity to control for this variable in the analysis.

## 5. Conclusions

Post-cardiac arrest syndrome may lead to hemodynamic instability caused by multiplex factors, including the effects of TTM. Proper hemodynamic monitoring and management of hemodynamic parameters are indispensable in this patient group. The accuracy of the PiCCO™ monitoring system was confirmed in the lower-temperature environment of post-cardiac arrest therapy. However, little is known about the effectiveness of PiCCO™ regarding mortality outcomes in post-cardiac arrest patients. Our analysis showed that while there was an interaction effect between PiCCO™-guided therapy, patients condition, and mortality, after 30 days for most conditions, and after 1 year, we saw either no effect of PiCCO™ monitoring on survival or a worsening of survival among patients who had no underlying conditions and received PiCCO™. Given the exploratory nature of our study, further investigations are needed to clarify which patients benefit from PiCCO™ monitoring during post-cardiac arrest therapy.

## Figures and Tables

**Table 1:** Patient characteristics. AMI: acute myocardial infarction; BLS: basic life support; CPR: cardiopulmonary resuscitation; EF: ejection fraction; IABP: intra-aortic balloon pump IHCA: in-hospital cardiac arrest; IQR: interquartile range; MI: myocardial infarction; n: number of patients; NSTEMI: non-ST-elevation myocardial infarction; PCI: percutaneous coronary intervention; PEA: pulseless electrical activity; ROSC: return of spontaneous circulation; STEMI: ST-elevation myocardial infarction; VF: ventricular fibrillation; and VT: ventricular tachycardia.

Patient Characteristics	Total
	n (%)
	or
	Median (IQR)
Total	63 (100%)
Age	64 (56, 69)
Gender (female in %)	19 (30%)
IHCA	11 (17 %)
Prior history:	
Hypertension	45 (71%)
Diabetes	18 (29%)
Hyperlipidemia	30 (48%)
AMI	15 (24%)
Stroke	6 (10%)
Circumstances of CPR:	
Patient on monitor when collapsed	9 (14%)
BLS performed by bystanders	49 (78%)
Time to ROSC (minutes)	20 (15, 30)
Initial rhythm:	
VF	42 (67%)
VT	2 (3%)
PEA	10 (16%)
Asystole	9 (14%)
Cause of cardiac arrest:	
STEMI	38 (60%)
NSTEMI	8 (13%)
Cardiac condition after ROSC:	
Cardiogenic shock (clinical signs)	14 (22%)
EF after ROSC (%)	36 (29, 48)
Therapy after ROSC:	
Catecholamine therapy	39 (62%)
Acute PCI	51 (81%)
Levosimendan	7 (11%)
IABP use	16 (25%)
Time to reach target temperature (hours)	3,8 (2.0, 5.1)
PiCCO™ application rate	33 (52%)
Died at 30 days	24 (38%)
Died at 1 year	36 (57%)

## Data Availability

All data generated and analyzed during this study are available from the corresponding author on reasonable request.

## Supplementary Materials

The following are available online at https://www.mdpi.com/article/10.3390/ijerph18105223/s1, Figure S1. Changes of temperature during target temperature management in PiCCO™ and non-PiCCO™ groups. Figure S2. Changes of heart rate during target temperature management in PiCCO™ and non-PiCCO™ groups. Figure S3. Changes of mean arterial pressure (MAP) during target temperature management in PiCCO™ and non-PiCCO™ groups. Figure S4. Changes of cardiac index, systemic vascular resistance index, global ejection fraction, and extravascular lung water index during target temperature management in PiCCO™ group. Figure S5. Changes of serum lactate level during target temperature management in PiCCO™ and non-PiCCO™ groups. Figure S6. Changes of dopamine dosage during target temperature management in PiCCO™ and non-PiCCO™ groups. Figure S7. Changes of dobutamine dosage during target temperature management in PiCCO™ and non-PiCCO™ groups. Figure S8. Changes of noradrenaline dosage level during target temperature management in PiCCO™ and non-PiCCO™ groups. Table S1. Characteristics of patients and initial therapy regarding PiCCO™ use, 30 day and 1 year mortality.
