# Supplementary material for: An Interaction Effect Analysis of Thermodilution-Guided Hemodynamic Optimization, Patient Condition, and Mortality after Successful Cardiopulmonary Resuscitation
Source: Int J Environ Res Public Health. 2021 May 14;18(10):5223. doi: 10.3390/ijerph18105223 (PMC8156244; doi:10.3390/ijerph18105223)
Supplement: Supplementary file 1 [file ijerph-18-05223-s001.zip › ijerph-1157852-supplementary.pdf]

An Interaction Effect Analysis of Thermodilution-Guided Hemodynamic Optimization, Patient Condition, and Mortality after Successful Cardiopulmonary Resuscitation

Supplementary material

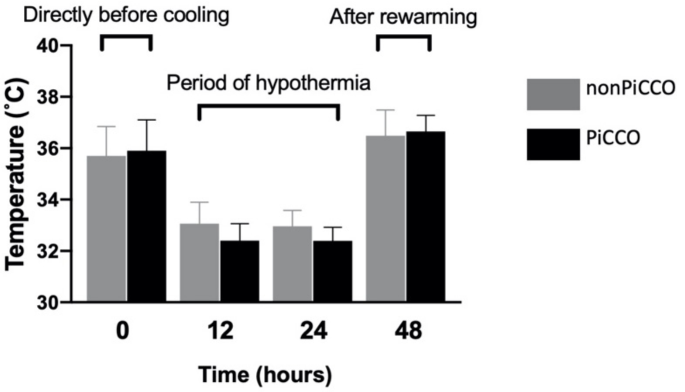

Figure S1. Changes of temperature during target temperature management in PiCCO™ and nonPiCCO™ groups.

0: initiation of cooling; 12: 12th hour of hypothermia; 24: 24th hour of hypothermia; 48: 48 hours after the initiation of cooling. Values are presented as median with interquartile range.

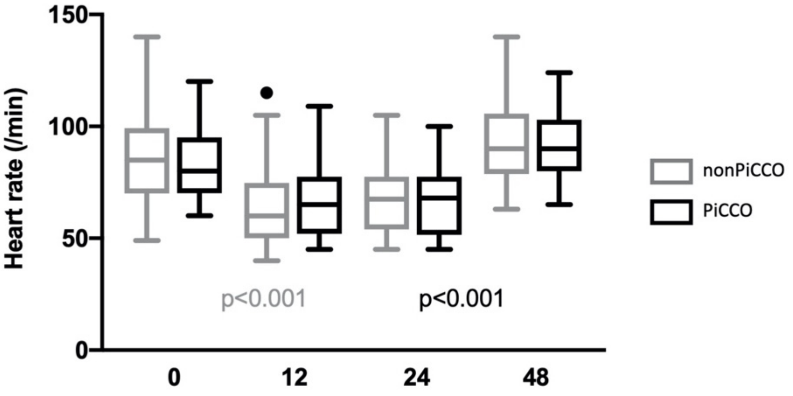

Figure S2. Changes of heart rate during target temperature management in PiCCO™ and nonPiCCO™ groups.

0: initiation of cooling; 12: 12th hour of hypothermia; 24: 24th hour of hypothermia; 48: 48 hours after the initiation of cooling. P values show the changes of heart rate during TTM within nonPiCCO™ (grey) and PiCCO™ (black) group (Friedman test). Mann-Whitney test was performed to compare PiCCO™ and nonPiCCO™ groups. Box-Whisker plot: Values are presented as Box-Whisker plot by Tukey method.

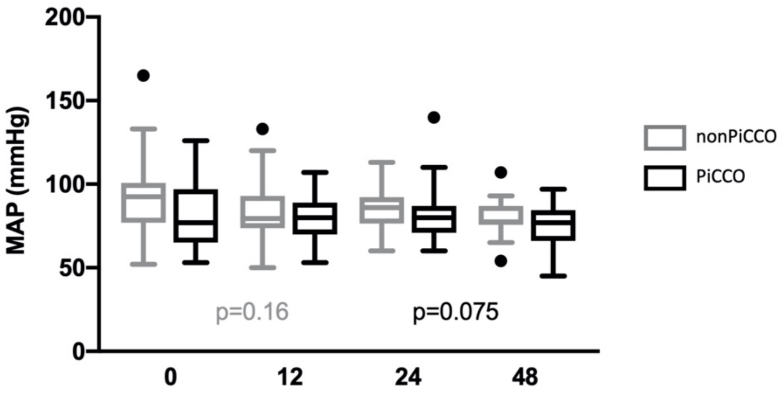

Figure S3. Changes of mean arterial pressure (MAP) during target temperature management in PiCCO™ and nonPiCCO™ groups.

0: initiation of cooling; 12: 12th hour of hypothermia; 24: 24th hour of hypothermia; 48: 48 hours after the initiation of cooling. P values show the changes of MAP during TTM within nonPiCCO™ (grey) and PiCCO™ (black) group (Friedman test). Mann-Whitney test was performed to compare PiCCO™ and nonPiCCO™ groups. Box-Whisker plot: Values are presented as Box-Whisker plot by Tukey method.

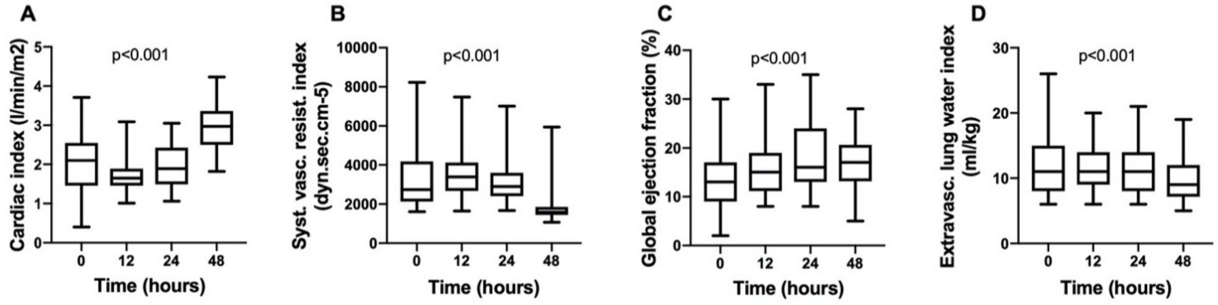

Figure S4. Changes of cardiac index, systemic vascular resistance index, global ejection fraction and extravascular lung water index during target temperature management in PiCCO™ group.

0: initiation of cooling; 12: 12th hour of hypothermia; 24: 24th hour of hypothermia; 48: 48 hours after the initiation of cooling. P values show the changes of hemodynamic parameters during the different phases of target temperature management (Friedman test).

Box-Whisker plot: the box extends from the 25 to 75 percentile and interprets median, while the whiskers show minimum and maximum values.

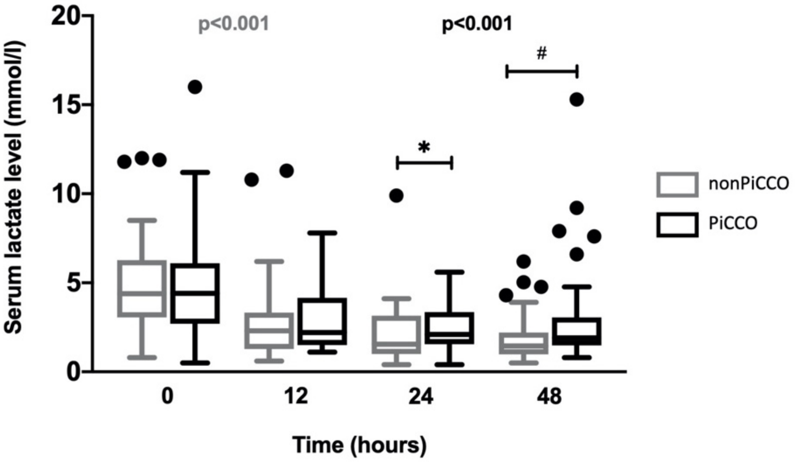

Figure S5. Changes of serum lactate level during target temperature management in PiCCO™ and nonPiCCO™ groups.

0: initiation of cooling; 12: 12th hour of hypothermia; 24: 24th hour of hypothermia; 48: 48 hours after the initiation of cooling. P values show the changes of serum lactate level during TTM within nonPiCCO™ (grey) and PiCCO™ (black) group (Friedman test). Values are presented as Box-Whisker plot by Tukey method. Mann-Whitney test was performed to compare PiCCO™ and nonPiCCO™ groups (✱: $p<0.05$; #: $p>0.05$ and <0.2).

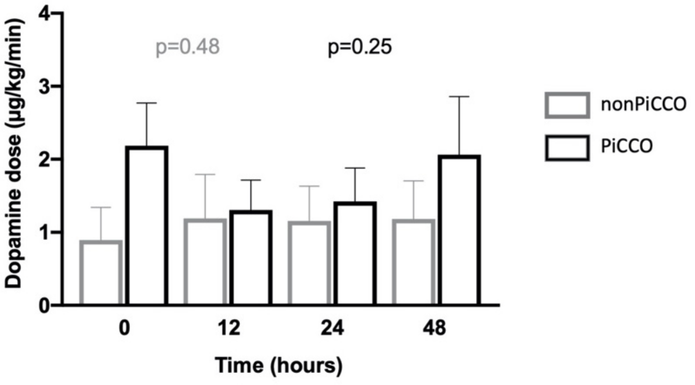

Figure S6. Changes of dopamine dosage during target temperature management in PiCCO™ and nonPiCCO™ groups.

0: initiation of cooling; 12: 12th hour of hypothermia; 24: 24th hour of hypothermia; 48: 48 hours after the initiation of cooling. P values show the changes of dopamine doses during TTM within nonPiCCO™ (grey) and PiCCO™ (black) group (Friedman test). Values are presented as mean and standard error of mean. Mann-Whitney test was performed to compare PiCCO™ and nonPiCCO™ groups.

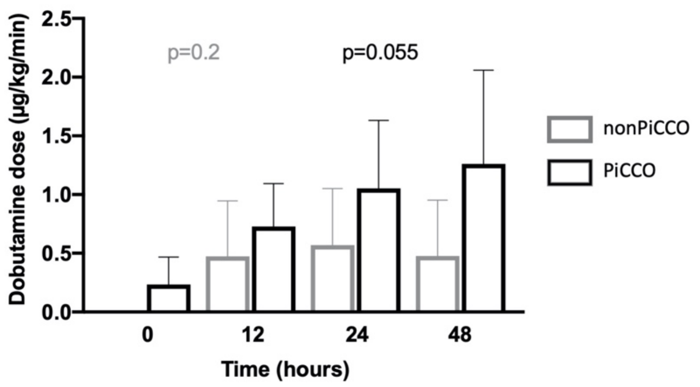

Figure S7. Changes of dobutamine dosage during target temperature management in PiCCO™ and nonPiCCO™ groups.

0: initiation of cooling; 12: 12th hour of hypothermia; 24: 24th hour of hypothermia; 48: 48 hours after the initiation of cooling. P values show the changes of dobutamine doses during TTM within nonPiCCO™ (grey) and PiCCO™ (black) group (Friedman test). Values are presented as mean and standard error of mean. Mann-Whitney test was performed to compare PiCCO™ and nonPiCCO™ groups.

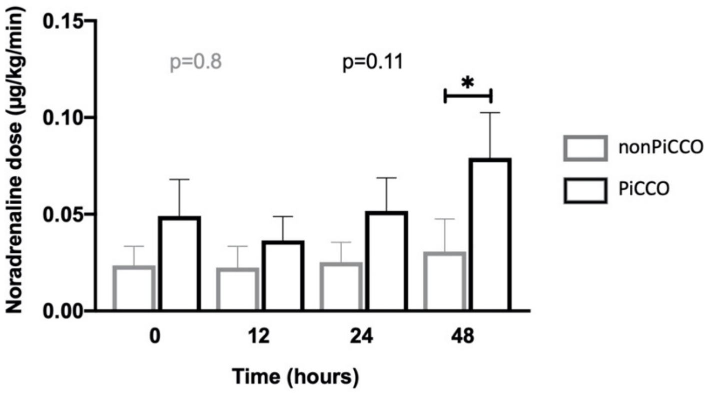

Figure S8. Changes of noradrenaline dosage level during target temperature management in PiCCO™ and nonPiCCO™ groups.

0: initiation of cooling; 12: 12th hour of hypothermia; 24: 24th hour of hypothermia; 48: 48 hours after the initiation of cooling. P values show the changes of noradrenaline doses during TTM within nonPiCCO™ (grey) and PiCCO™ (black) group (Friedman test). Values are presented as mean and standard error of mean. Mann-Whitney test was performed to compare PiCCO™ and nonPiCCO™ groups (✱: $p<0.05$).

Table S1. Characteristics of patients and initial therapy regarding PiCCO® use, 30 day and 1 year mortality.

	Total n (%) or Median (IQR)	nonPiCCO Group n (%) or Median (IQR)	PiCCO Group n (%) or Median (IQR)	*p*	Alive at 30d n (%) or Median (IQR)	Died at 30d n (%) or Median (IQR)	*p*	Alive at 1y n (%) or Median (IQR)	Died at 1y n (%) or Median (IQR)	*p*
Total	63 (100%)	30 (100%)	33 (100%)		39 (100%)	24 (100%)		27 (100%)	36 (100%)	
Age	64 (56, 69)	64 (56, 71)	65 (56, 69)	1.000	64 (56, 71)	65 (55, 69)	0.773	64 (53, 70)	65 (57, 69)	0.576
Gender (female in %)	19 (30%)	13 (43%)	6 (18%)	**0.030**	27 (69%)	17 (71%)	0.893	19 (70%)	25 (69%)	0.937
IHCA	11 (17 %)	2 (7%)	9 (27%)	**0.030**	6 (15%)	5 (21%)	0.409	5 (19%)	6 (17%)	0.553
Prior history:										
HT	45 (71%)	15 (50 %)	30 (91%)	**>0.001**	26 (67%)	19 (79%)	0.219	20 (74%)	25 (69%)	0.687
DM	18 (29%)	8 (27%)	10 (30%)	0.750	13 (33%)	5 (21%)	0.219	8 (30%)	10 (28%)	0.872
HLP	30 (48%)	11 (37%)	19 (58%)	*0.100*	15 (38%)	15 (63%)	*0.064*	11 (41%)	19 (53%)	0.344
AMI	15 (24%)	5 (17%)	10 (30%)	*0.170*	9 (23%)	6 (25%)	0.862	5 (19%)	10 (28%)	0.55
Stroke	6 (10%)	0 (0%)	6 (18%)	**0.016**	6 (15%)	0 (0%)	**0.048**	3 (11%)	3 (8%)	1.000
Circumstances of CPR:										
Patient on monitor when collapsed	9 (14%)	2 (7%)	7 (21%)	*0.090*	5 (13%)	4 (17%)	0.470	2 (7%)	7 (19%)	0.279
BLS performed by bystanders	49 (78%)	23 (77%)	26 (79%)	0.840	33 (85%)	16 (67%)	*0.096*	24 (89%)	25 (69%)	*0.077*
Time to ROSC (minutes)	20 (15, 30)	20 (11, 30)	20 (16, 33)	0.770	20 (12, 30)	20 (18, 34)	*0.173*	17 (11, 30)	20 (20, 34)	*0.056*
Initial rhythm:										
VF	42 (67%)	20 (67%)	22 (67%)	1.000	30 (77%)	12 (50%)	**0.028**	23 (85%)	19 (53%)	**0.008**
VT	2 (3%)	0 (0%)	2 (6%)	0.270	1 (3%)	1 (4%)	0.620	0 (0%)	2 (6%)	0.502
PEA	10 (16%)	6 (20%)	4 (12%)	0.300	6 (15%)	4 (17%)	0.579	3 (11%)	7 (19%)	0.494

Asy	9 (14%)	4 (13%)	5 (15%)	0.560	2 (5%)	7 (29%)	**0.012**	1 (4%)	8 (22%)	*0.066*
Cause of cardiac arrest										
Rate of STEMI	38 (60%)	14 (47%)	24 (73%)	**0.035**	26 (67%)	12 (50%)	*0.189*	19 (70%)	19 (53%)	*0.158*
Rate of NSTEMI	8 (13%)	5 (17%)	3 (9%)	0.300	5 (13%)	3 (13%)	0.645	3 (11%)	5 (14%)	1.000
Cardiac condition after ROSC										
Rate of cardiogenic shock (clinical signs)	14 (22%)	4 (13%)	10 (30%)	*0.090*	4 (10%)	10 (42%)	**0.005**	1 (4%)	13 (36%)	**0.002**
EF after ROSC (%)	36 (29, 48)	36 (30, 50)	35 (27, 45)	0.420	36 (30, 48)	36 (26, 47)	0.486	36 (29, 48)	36 (28, 47)	0.809
Therapy after ROSC:										
Catecholamine therapy	39 (62%)	14 (47%)	25 (76%)	**0.018**	18 (46%)	21 (88%)	**0.001**	11 (41%)	28 (78%)	**0.003**
Acute PCI	51 (81%)	22 (73%)	29 (88%)	*0.130*	34 (87%)	17 (71%)	*0.109*	23 (85%)	28 (78%)	0.531
Levosimendan	7 (11%)	2 (7%)	5 (15%)	0.250	4 (10%)	3 (13%)	0.543	2 (7%)	5 (14%)	0.689
IABP use	16 (25%)	3 (10%)	13 (39%)	**0.007**	6 (15%)	10 (42%)	**0.019**	3 (11%)	13 (36%)	**0.039**
Time to reach target temperature (hours)	3,8 (2.0, 5.1)	4.0 (2.8, 5.1)	3.8 (2.0, 5.0)	0.350	4.0 (2.5, 5.1)	3.8 (2.0, 5.1)	0.569	4.0 (3.0, 5.1)	3.8 (1.3, 5.1)	0.254

Significant differences are expressed. Mann-Whitney`s U test and Chi-square test (or Fisher`s exact test in cases of small sample size) are applied. Bold *p*-values: $p < 0.05$, italic *p*-values: $p < 0.2$ and $p > 0.05$. alive at 30d: patients who survived 30 days; died at 30d: patients who did not survive first 30 days after ROSC; alive at 1y: patients who survived one year; died at 1y: patients who did not survive one year after ROSC; AMI: acute myocardial infarction; Asy: asystole; BLS: basic life support; CPR: cardiopulmonary resuscitation; DM: diabetes mellitus; HLP: hyperlipidemia; HT: hypertension; IABP: intra-aortic balloon pump; IHCA: in-hospital cardiac arrest; n: number of patients; NSTEMI: non-ST segment elevation myocardial infarction; OHCA: out-of-hospital cardiac arrest; PCI: percutaneous coronary intervention; PEA: pulseless elctrical activity; PiCCO: Pulse index Continuous Cardiac Outpit; ROSC: return of spontaneous circulation; SD: standard deviation; STEMI: ST segment elevation myocardial infarction; VF: ventricular fibrilation; VT: ventricular tachycardia.
